# Development of the multi-directional ablation process using the femtosecond laser to create a pattern on the lateral side of a 3D microstructure

**DOI:** 10.1038/s41598-023-32030-8

**Published:** 2023-03-23

**Authors:** Cheol Woo Ha, Yong Son

**Affiliations:** Korea Additive Manufacturing Innovation Centre (KAMIC), Korea Institute of Industrial Technology (KITECH), Siheung-si, Republic of Korea

**Keywords:** Laser material processing, Lithography, Nonlinear optics, Ultrafast photonics

## Abstract

Two-photon stereolithography (TPS) is widely used for the fabrication of various three–dimensional (3D) structures with sub-micron fabrication resolution in a single fabrication process. However, TPS is unsuitable for microstructures with fine-hole patterns. The laser ablation process can be easily drilled, or made holes in various materials. However, in the case of laser ablation, the focal plane of the laser is fixed, which is limited to the processing plane. In this study, a multidirectional ablation process is studied to apply laser ablation to various processing planes of a 3D microstructure fabricated by the TPS process. A 3D hybrid fabrication process with the advantages of both TPS and laser ablation is expected to improve the fabrication efficiency. The 3D hybrid process is proposed based on a single laser source. The microstructure is fabricated using TPS, and the multi-directional ablation process creates a hole in the lateral side of the 3D microstructure. To develop the multidirectional ablation process, the reflecting mirror system should be designed to adaptably rotate the laser focal plane and guide the laser path for the target process plane. Through various examples, we demonstrate the ability of the multi-directional ablation process with various examples.

## Introduction

Recently, the need for an effective fabrication process related to nanotechnology (NT), biotechnology (BT), and information technology (IT) has significantly grown in the development of 3D nano/microdevices and highly integrated systems. Micro- and nanofabrication techniques include soft lithography^1,2^, photolithography^3,4^, and etching^5,6^ using a combination of these techniques, and various nano-/micro-systems have been fabricated. Advanced technology for the more complex design of structures, holographic lithography^7,8^, self-assembly^9,10^, and laser direct writing^11–13^ have been employed. Particularly, laser direct writing has significant advantages for the fabrication of three-dimensional structures because the laser scanning path is controlled according to three-dimensional computer-aided design (CAD) data. Laser direct writing involves two methods: additive and subtractive methods.

Direct writing using a femtosecond laser (DWFL) is an effective 3D nano/micro-process. DWFL is a maskless, simple, and cost-effective method for fabricating 2D and 3D nano/microstructures. The additive process of DWFL (i.e., two-photon stereolithography process; TPS) has strong merits for the direct fabrication of 2D and 3D microstructures with sub-micron resolution^14–17^. However, TPS has some limitations. For example, the resolution and fabrication speed of the additive process are insufficient for nanoscale applications^18,19^. Some of these limitations can be ameliorated using the subtractive process of DWFL. A typical subtractive process is ablation using a focused high-power laser. The ablated micropattern can be written using laser scanning according to the designed path.

Several studies have been conducted on hybrid fabrication methods, which have the advantages of both additive and subtractive processes. For example, the limited fabrication resolution of the additive process can be improved using a subtractive process^20^. Using the TPS process, a microstructure with a hole or small gap is difficult to fabricate. A small hole or gap tends to solidify and block owing to the superposition of the laser powers^21,22^. Additionally, the ineffective laser scanning path for the three-dimensional structure causes a long fabrication tim^23,24^. However, the laser ablation process is suitable for creating holes in structures. Therefore, by employing the hybrid fabrication process of TPS and laser ablation, the three-dimensional nano-/micro-structure can be effectively fabricated with higher precision and a shorter fabrication time.

In this study, the concept of a 3D hybrid process is based on a single laser source. The 3D hybrid process employs the strengths of both additive and subtractive processes in the DWFL technique. As one of the 3D hybrid processes, a multi-directional ablation process is proposed. Laser ablation is generally performed using a vertically exposed laser according to the substrate. However, when a microstructure such as a micro-pinhole or micro-slit, including a hole or slit on the side of the 3D microstructure, should be fabricated, it is necessary to change the travel direction of the exposed laser to process at various processing surfaces. This paper showed that it is possible to fabricate nano-sized holes using the laser ablation process as a post-process. Generally, the TPS process's fabrication resolution is about 200 nm. However, for a hole structure that is not a single line or open cell structure, it is possible to fabricate a hole of the size of about 1um or more based on the TPS process. Because the TPS process is the additive polymerization process, the weakly polymerization area is overlapped, and the superposition of polymerization leads to full cross-liking. The degree of weak polymerization will vary depending on the material. but in the case of general photocurable resin, the hole would be fabricated without overlapping effect only when the distance is at least 1um. The TPS process is based on photolithography, and various photocurable polymers can be used for the TPP process. The hybrid process is when a photocurable polymer is cured through TPP, and then a laser ablation process is performed as the post-processing. Among various photocurable polymers, the Su-8 material was used in the TPP process in this study. Because Su-8 material has good mechanical properties and is advantageous for high aspect-ratio structures, the Su-8 material is widely used for various applications based on the TPP process. The reflecting mirror was designed to guide the travel direction of the laser, considering the main specifications of the femtosecond laser writing system, and the ablation characteristics as a function of the working distance (the distance between the reflecting mirror and structure) were studied. The reflecting mirror was fabricated by MEMS processes, such as UV lithography, wet etching, and CVD. The effectiveness of the proposed process is demonstrated in several applications, including micro pinholes and microtubes with micro holes in various directions.

## Materials and methods

### Materials

All chemicals utilized in this study were procured from Sigma-Aldrich, unless mentioned otherwise. The two-photon photopolymerizable resist, Su-8 2035 (Microchem Co.) was used for all fabrications. 1 g of SU-8 2035 was sensitized by adding 2 mg of a highly efficient phenylene vinylene bridged TPA dye. The Su-8 was prebaked at 95 °C for 10 min before TPS process. After fabrication, the Su-8 was postbaked at 95 °C for 10 min. The fabricated 3D microstructure was developed by propylene glycol monomethyl ether acetate (PGMEA).

### Fabrication process based on DWFL

#### Two-photon lithography for additive process

Most of the photocurable resin can be applied to the TPS process. The photocurable resin consist of monomer and a initiator for inititation of the cross-linking reaction. For effective cross-linking reaction, the inititor should be desined to adjust to the wavelength of the exposed light. The laser source of the TPS process is femtosecond laser. Unlike the single-photon absorption process, when a femtosecond laser is exposed to a photocurable resin, the photocurable resin simultaneously absorb two photons to the excited state. Subsequently, the absorbed energy is released and returns to the ground state. Owing to two-photon absorption, the tiny volume at the focus of the laser can polymerize the photocurable resin and is called as a voxel (volume of the pixel). The TPS process based on two-photon absorption was used to fabricate the 3D microstructure with a sub-micron fabrication resolution^14–17^. However, it is necessary to evaluate the TPS processes that can improve the fabrication resolution or processing speed. A hybrid fabrication process with TPS and laser ablation can improve the fabrication efficiency of the TPS process.

A two-photon lithography system was developed based on a previous study. A femtosecond laser with a wavelength of 780 nm, a pulse frequency of 80 MHz, and a pulse duration of 100 fs was used. An oil-immersion objective (Numerical Aperture, NA = 1.4, × 100, immersion oil used, Olympus) was used to focus the laser beam. The epoxy photoresist Su-8 2035 was used for all the microfabrications. The glass substrate had dimensions of 30 × 40 × 0.7 mm^3^. The x, y, and z directions were scanned using piezoelectric stages (P-622 for z-stage, P-628 for xy stage Physik Instrumente (PI)) with a resolution of 10 nm. After fabrication, 3D microstructures were developed in propylene glycol monomethyl ether acetate (PGMEA) for 10 min. The 3D microstructures were then rinsed in an isopropyl alcohol (IPA) bath for 1 min. All the processes were performed at room temperature.

#### Femtosecond laser ablation for subtractive process

When a pulsed laser, such as a femtosecond laser, is exposed on the sample surface, the energy of the laser causes the vibration of free electrons in the sample material^25,26^.

This vibration is delivered to the lattice of the material, resulting in a rapid increase in temperature, and heat accumulation is generated. The resolution of the laser ablation process is affected by the heat accumulation. The surface particles under high temperature and pressure are transformed into steam and plasma forms and are removed. This phenomenon is known as laser ablation. The time when the heat generated by the laser spread in the substrate was known as up to several nanoseconds. In the laser ablation process with the nanosecond laser, the typical 10 µm ~ 1 mm width heat effect zone (HAZ) is usually observed. In the case of the femtosecond laser, the penetration length of thermal diffusion in the material is limited because the pulse duration is shorter than the heat accumulation speed. Therefore, the laser ablation process with the femtosecond laser leads to very small or almost free HAZ, so the ultra-precision process is possible^27,28^. Therefore, femtosecond laser is powerful tool for precise materials processing such as thin film patterning^29^, wafer dicing^30^, micro fluidic channel^31^, etc.

In this study, laser ablation was performed using the same laser system as that used for two-photon lithography. For the TPS process, the laser power was in the range of 10 ~ 100 mW, whereas for the laser ablation process, the laser power was greater than 100 mW. For the ablation process, the laser was scanned using a galvano scanner (LightningTM II, Novanta Photonics).

Since the optics used for the laser ablation part is not an F-theta lens, the focal plane is not planar, and therefore, it will strongly limit the irradiation area where the minimum beam spot can be focused. The process area of the laser ablation system used in this paper is limited to 400 µm. The ablation resolution is almost similar within this process area. For the TPS process, the microstructure is possible to fabricate within 500um size according to the travel range of the piezoelectric stage. However, in this paper, the size of the microstructure fabricated by the two-photon lithography is less than 50 µm. For the hybrid process proposed in this paper, the microstructure is fabricated using TPS, and then the ablation process creates a hole in the 3D microstructure. Therefore, even though the ablation system is not used the F-theta lens, the size of the microstructure is within the process area, and it is enough to apply the ablation process to the microstructure fabricated by the TPS process.

### Morphological characterization

The micro 3D structures were platinum (Pt) sputtered coating and imaged via SEM (FE-SEM; NNS-450, FEI Hong Kong Company). A secondary electron detector visualized all SEM images with an accelerating voltage of 1.0 ~ 1.5 kV in a vacuum. SEM combined with energy dispersive analysis of X-rays (EDAX).

## Results

### Conceptual design of hybrid system

A hybrid system with the advantages of both TPS and laser ablation is expected to improve the efficiency of existing microfabrication processes. Particularly, it is necessary to study TPS processes that can improve the fabrication resolution or processing speed using a TPS hybrid and other existing subtractive processes. Moreover, in the case of a simple hole pattern, laser ablation is more appropriate than TPS. When considering the polymerizing mechanism of photocurable resin, the cross-linking tends to be easily spread by the surrounding energy; therefore, it is challenging to express the micro-hole pattern using the TPS process.

**Figure 1:**
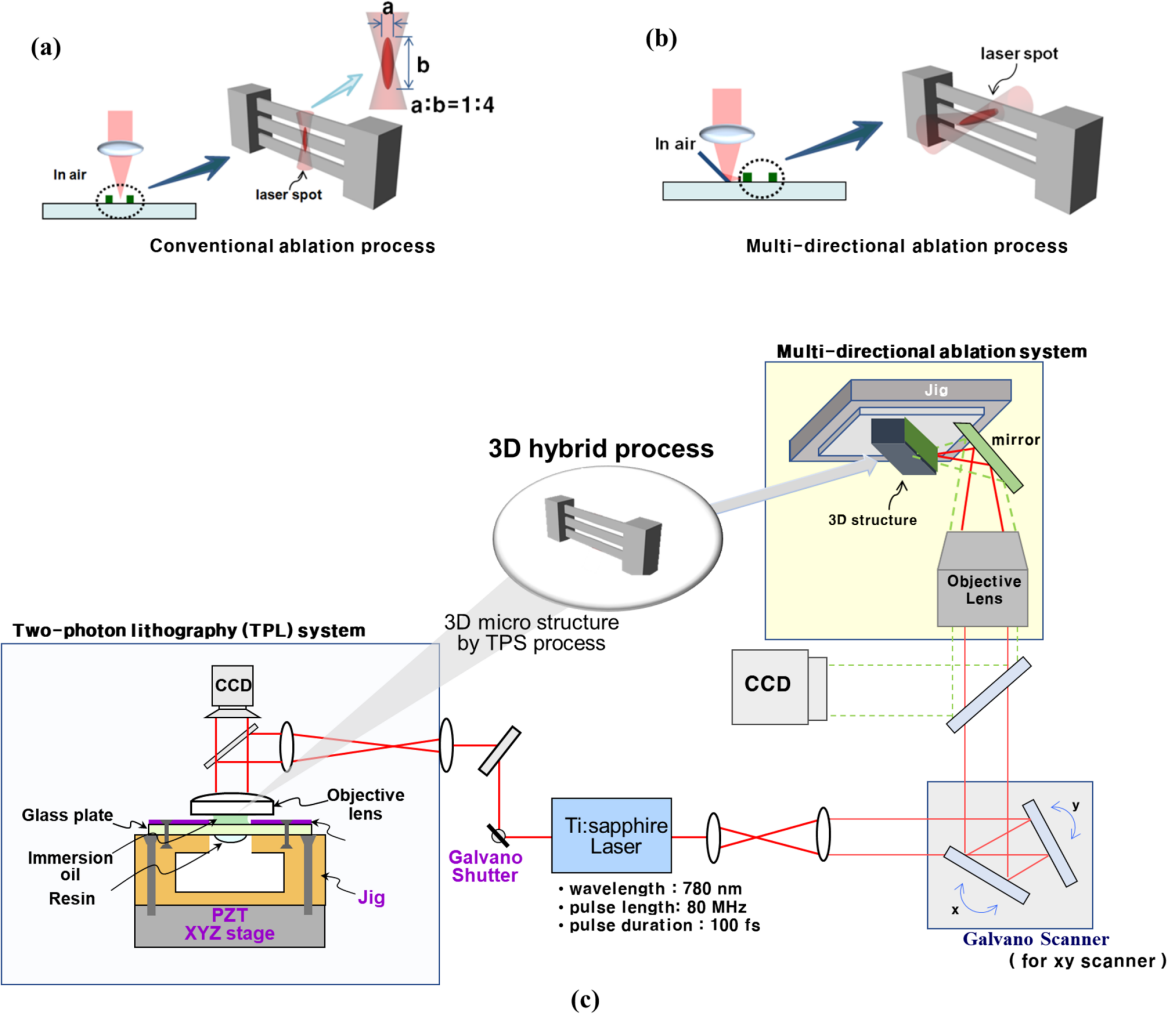
(**a**) Schematic diagram of (**a**) conventional laser ablation process in the vertical direction and (**b**) multi-directional ablation process, which controls the direction of the laser path. (**c**) Diagram showing the 3D hybrid process employing TPS system and multi-directional ablation system. Laser ablation can improve a 3D structure fabricated by the two-photon stereolithography process.

However, in the conventional laser ablation process, the focal plane of the laser is fixed, which is limited to the processing plane. Because the unit ablation volume in the laser focus is long in the longitudinal direction and short in the lateral direction, it is unsuitable for lateral surface patterning. For example, when a multi-beam structure needs to be drilled in a single beam structure by a conventional ablation process, it cuts the entire beam structure, or ablates the beam with a long-elliptical pattern (Fig. 1a). If the processing plane can be adaptably rotated by changing the focal plane of the laser, the laser ablation process can be applied in various directions, without restrictions on the direction of the processing plane.

In this study, we propose a 3D hybrid microfabrication system that has the advantages of both additive and subtractive processes. The proposed hybrid system can be used to fabricate 3D microstructures effectively. To overcome the problem of the process direction of the conventional laser ablation process, a multidirectional ablation process was proposed by controlling the focal plane of the femtosecond laser. For drilling in a multibeam structure, the ablation resolution of the rotated laser can produce a precise hole pattern in the single-beam structure (Fig. 1b). Figure 1c shows a schematic of the multidirectional ablation system. The multidirectional ablation system was based on an existing TPS system. A Ti: sapphire femtosecond laser was used as the laser source. The laser was scanned using a Galvano scanner. All processes were monitored using a CCD camera. The laser was reflected by a mirror and reached the lateral surface of the microstructure.

### Element design for the multi-directional ablation system

**Figure 2:**
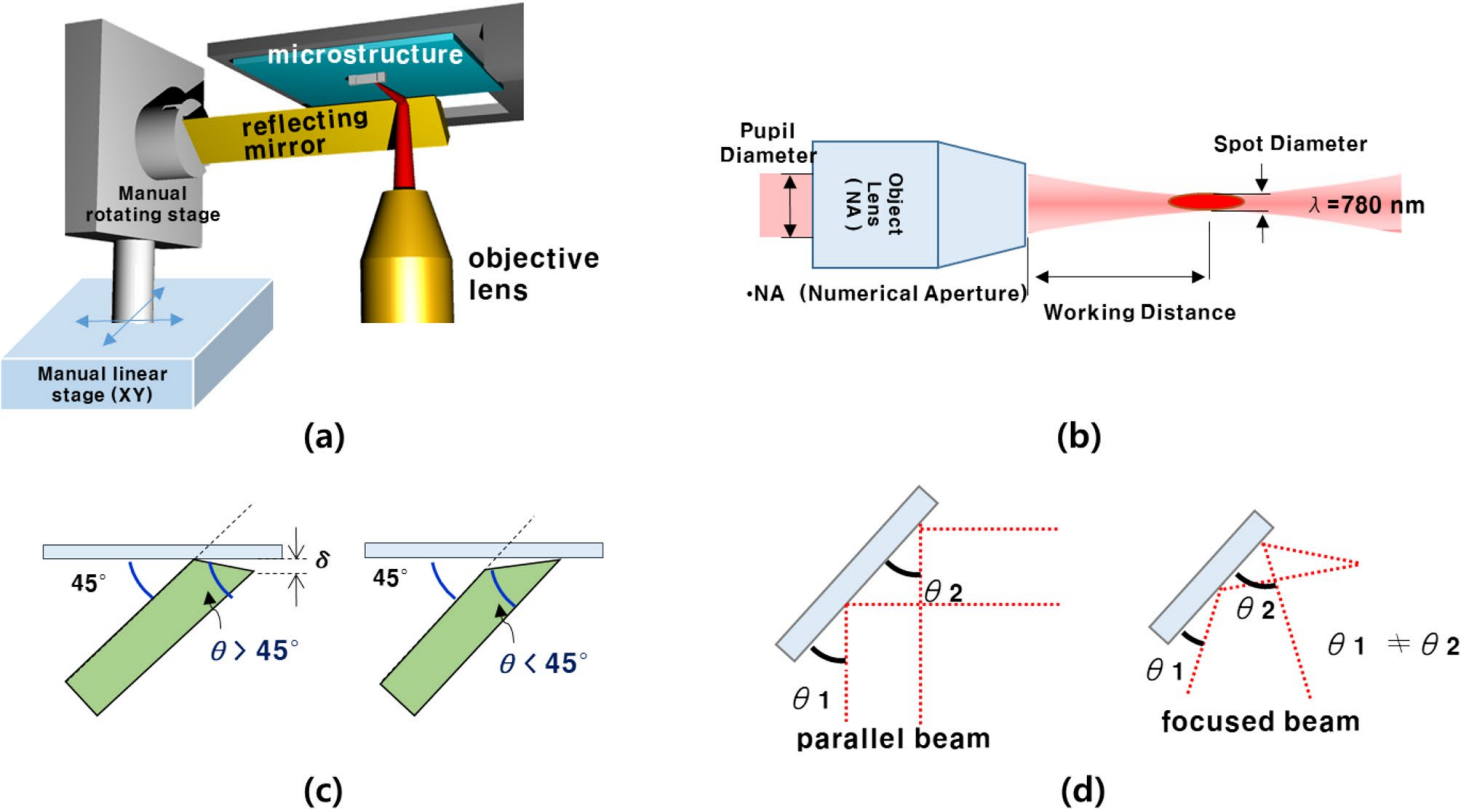
(**a**) Schematic diagram of the mirror system, which consists of a gold-coated mirror and NA 0.3 objective lens. The mirror reflects a femtosecond laser to change the beam. (**b**) Schematic illustration of the objective lens structure. (**c**) Design condition of reflection mirror. (**d**) Schematic illustration of the focused beam.

Figure 2a shows the conceptual design of a reflecting mirror system for guiding the focal direction of the laser. The reflecting mirror for guiding the laser path is a key element in the multidirectional ablation process. The position of the reflecting mirror is manually controlled by linear stage and rotating stage. The reflecting mirror is approaching to the microstructure by linear stage and it control the angle of the reflecting mirror by rotating stage. The reflecting mirror is fixed to the rotating stage. The direction of the incident laser was changed using the reflecting mirror. The ablation-processing plane was also changed and controlled by the reflecting mirror. The laser was reflected by the mirror and was designed to be processed on the side. Thus, the focal plane of the laser and the processing plane of the laser ablation can be controlled by the mirror.

The laser system for the multidirectional ablation process was developed using a proper objective lens and a reflecting mirror for controlling the laser focal plane. An objective lens was employed to focus the laser beam. Figure 2b shows various parameters of the objective lens. The pupil diameter refers to the diameter of the cross section of the laser when the laser exits the object lens. The mirror surface should be designed to be larger than the pupil diameter to allow laser reflection without laser loss. The working distance refers to the distance between the objective lens and laser focus. If the working distance is too short, it is difficult to set up a mirror between the objective lens and sample structure. The spot diameter refers to the diameter of the laser cross-section at the laser focus. The spot diameter was related to the ablation resolution of the focused laser. The Numerical Aperture (NA) value represents the amount of light entering the object. λ is the wavelength of the femtosecond laser, which was 780 nm in this study. The working distance should be long enough to obtain sufficient space where the mirror surface is installed between the object lens and laser focal.

Table 1 lists the pupil diameter, spot diameter, and working distance for the object lenses according to various NA values. In the case of NA 1.4, 0.75, and 0.5, the working distance has a short distance of less than a few mm, thus, it is unsuitable for the mirror system set-up because more space is needed for the reflecting mirror installation. The remaining alternatives of the NA value were 0.3 and 0.13. The spot diameter of NA 0.3 was smaller, and it is expected to be a more precise ablation process. Therefore, in this study, we opted to use an objective lens with an NA value of 0.3 to set up the multi-directional ablation process.

**Table 1:** Objective lens parameters with various NAs^32^.

Objective lens	Numerical aperture (NA)	Working distance (mm)	Pupil diameter (mm)	Spot diameter (µm)
Case 1	1.4	0.20	0.56	0.68
Case 2	0.3	10	4.42	3.17

### Fabrication of reflecting mirror

The multi-directional ablation process is required for the reflecting mirror to change the focal plane of the laser for the multi-directional ablation process.

The geometry of the mirror tip is related to the ablation process range. Figure 2c shows the design conditions of the reflecting mirror. The reflecting mirror is required to obtain a wide production range by improving the shape of the tip of the reflecting mirror. If the angle (θ) of the mirror is greater than 45°, the mirror tip is detached from the substrate. Because the mirror tip was far from the substrate by δ, the height of the laser ablation processing range was reduced by δ from the substrate. Moreover, there was a processing limit height in the target microstructure. When the angle of the mirror tip was less than 45°, the laser could be closely exposed to the substrate and could ablate the bottom part of the microstructure. Therefore, the angle of the mirror tip was designed to be less than 45° so that the mirror tip could be in contact with the substrate. Consequently, the laser ablation processing range was stably protected.

It is important to determine a coating material with a high reflectivity for the incident laser beam. Figure 2d shows the incident angle of the focused beam. The focused laser beam was reflected at various angles of incidence. During the ablation process, the galvano mirror was rotated to create a pattern in the x and y directions of the processing plane. Subsequently, the incident angle of the laser was changed during the ablation process. Therefore, coating materials must have a high reflectivity according to the various incident angles of the laser. Generally, high reflection (HR) coatings and gold coatings are well known for their high reflectivity for 780 nm lasers. However, the HR coating was strictly designed to have a high reflectance at an incident angle of 45°. In contrast, the gold coating exhibited a slight variation at various incident angles. Therefore, in this study, a reflecting mirror for multi-directional ablation was coated with gold using the PVD process.

**Figure 3:**
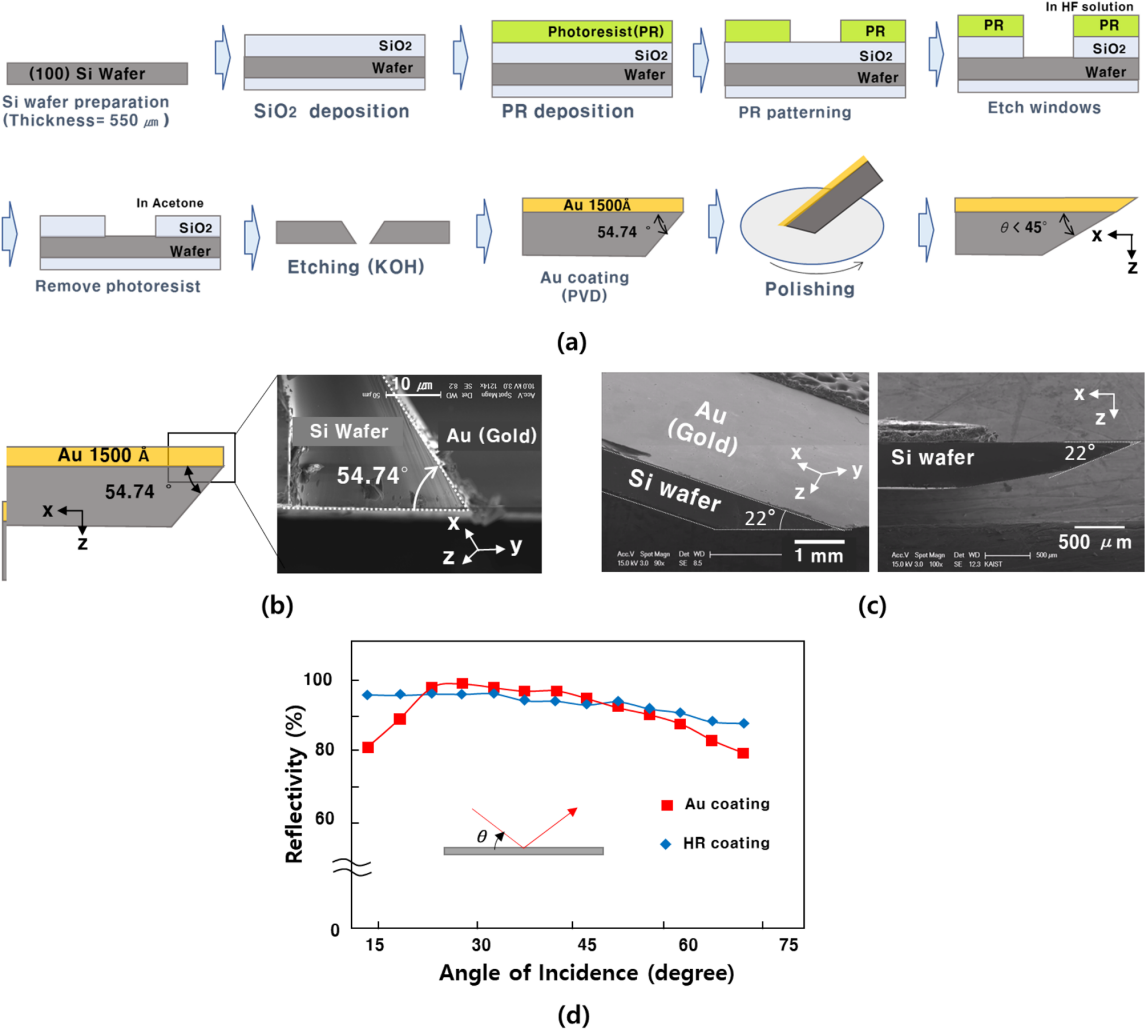
(**a**) Fabrication process of the reflecting mirror. The reflecting mirror is fabricated by a Si wafer with several steps. (**b**) SEM image of the gold-coated mirror. Before polishing the mirror tip, its angle is 54.74°. (**c**) SEM image of the gold-coated mirror. After polishing the mirror tip, its angle is 22°. This result satisfies the designed angle. (**d**) Reflectivities of the HR and gold-coated reflecting mirrors according to various mirror angles.

As discussed before, the reflecting mirror should be coated with gold, and the tip of the reflecting mirror should be designed under 45° or less. The fabrication process of the reflecting mirror is shown in Fig. 3a. The gold-coated mirror was fabricated using a potassium hydroxide (KOH) wet etching process. An SiO_2_ layer was used as the hard mask. An etching liquid (KOH) was used for the wet-etching process at 85 ℃ for 6 h. After the wet-etching process, because the etching speed in the wafer crystal surface (100) is much faster than the wafer crystal surface (111) direction, the angle of the wafer tip is 54.75°, as shown in Fig. 3b. Through the PVD process, the gold coating was fabricated with a thickness of 1500 Å. Additionally, the tip of the reflecting mirror was polished. As shown in Fig. 3c, the angle of the fabricated mirror tip was 22°, which satisfies the design conditions; the angle of the mirror tip was 45° or less.

We measured the reflectivities of the HR and gold coatings at various incident angles. In Fig. 3d, the gold-coated mirror has a lower reflectivity compared to the HR coating at an incident angle of 45°, but the reflectivity of the gold-coated mirror shows smaller deviations at various incident angles compared to the HR-coated mirror. The reflectivity of the gold-coated mirror was measured at 93.7% according to the incident angle at 45°.

### Evaluation of multi-directional ablation process

**Figure. 4:**
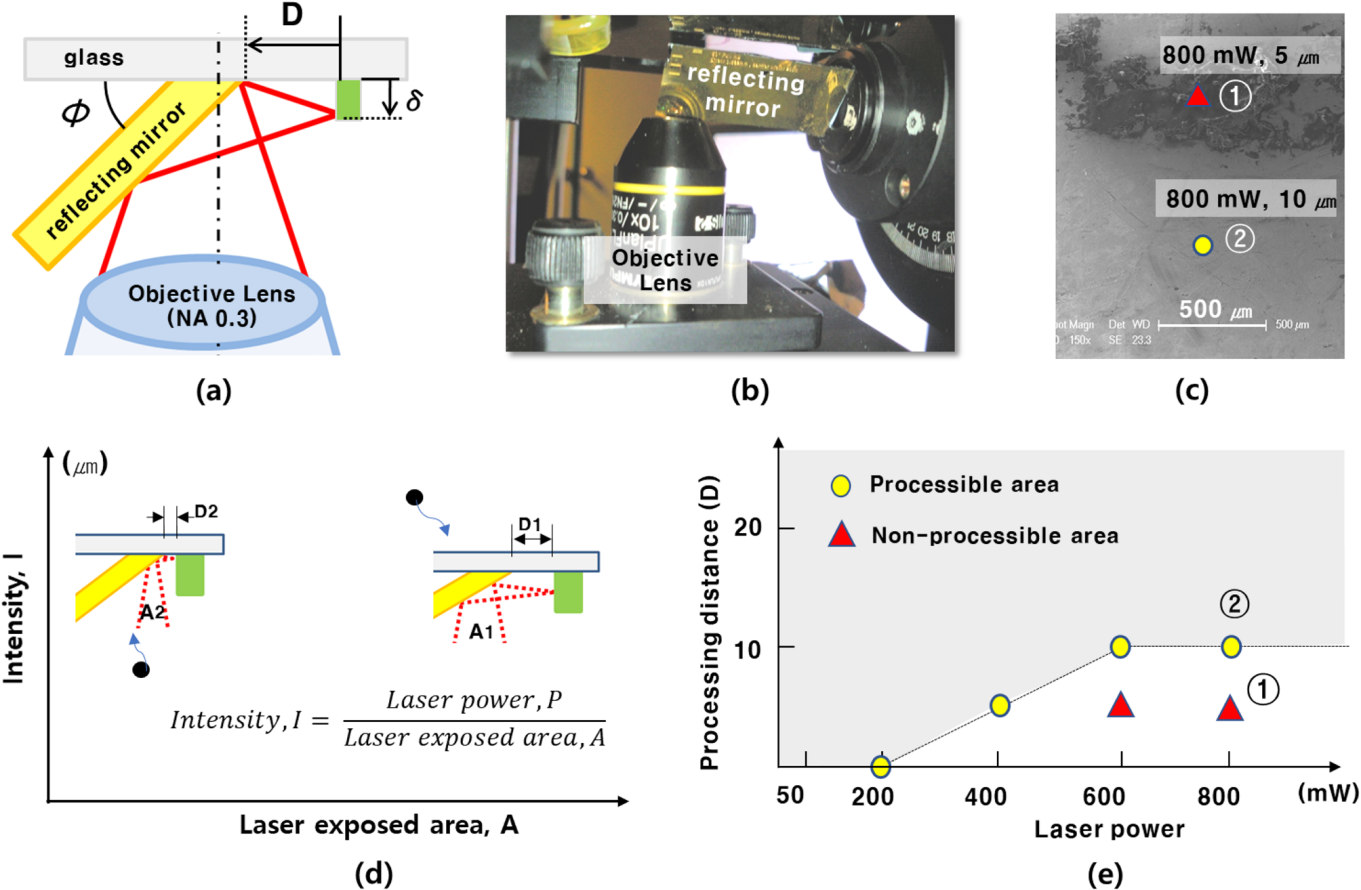
(**a**) Schematic illustration of laser reflecting in multi-directional ablation system. (**b**) Photo of the multi-directional ablation system. (**c**) Schematic graph of laser intensity according to laser exposed area. As the laser intensity (I) increases, the processing distance (D) is shortened. (**c**) SEM image showing the experimental results of the reflecting mirror surface damage test by various exposed laser powers with 800 mW at the processing distances of 5 μm and 10 μm. The red- and yellow-colored symbols indicate the point of EDAX analysis. (**d**) Fabrication distance between the reflecting mirror and structure considering the damage. (**e**) Processable area for multi-directional ablation process according to the laser power and process distance.

It is necessary to evaluate the processability of the multidirectional ablation process based on various design parameters. Figure 4a shows the design parameters of the reflecting mirror system. The “mirror rotation angle (Φ)” determines the processing plane of the multi-directional ablation process. In this study, the mirror rotation angle was fixed at 45° and the reflected laser was exposed to the lateral side of the vertical structure. The “processing distance (D)” reflects the distance from the reflecting mirror surface to the structure. Figure 4b shows the multidirectional ablation system. There are several references about gold patterning or damage by femtosecond laser^33,34^. It is important that the laser ablation process should be performed with a certain laser power within the proper laser power range so that the gold-coated reflecting mirror is not damaged. As shown in Fig. 4c, when the processing distance is too short, the exposed laser area in the reflecting mirror surface is narrowed, and the laser intensity is too strong to damage the reflecting mirror surface. However, if the processing distance is too long, it is difficult to expose the laser beam to the bottom of the structure. Therefore, there is a process-limited area from the bottom of the microstructure, where the laser is not exposed.

It is necessary to study the range of the processing distance so that the reflecting mirror surface is not damaged by the laser with a minimum process-limited area. By changing the laser power to 800 mW at 200 mW intervals and processing distances of 5 μm, 10 μm, and 15 μm, the reflecting mirror surface was analyzed by EDAX to determine whether the reflecting mirror surface was damaged by the laser. The laser was then irradiated for 10 s. Figure 4d shows the SEM image of the experimental results of the mirror surface damage test for various exposed laser powers. There were two points in the area damaged by the laser and non-damaged area. The red-colored triangular symbol in the damaged area indicates the EDAX analysis point, where the laser was exposed to 800 mW at a processing distance of 5 μm. The yellow-colored circular symbol in the non-damaged area indicates the EDAX analysis point, where the laser was exposed to 800 mW and a processing distance of 10 μm. In the non-processive area (①), it can be observed that the Si component is dominant on the mirror surface according to the EDAX data. This result indicates that the gold-coated surface layer was damaged by the laser exposure. In the processable area (②), the gold component was dominant. This indicates that the gold-coated surface layer was not damaged by the laser. Figure 4e shows the processable area according to the laser power and process distance. The yellow-dot indicated the processive point and the reflecting mirror is not damaged by the laser. However, the red-dot indicated the non-processive point and the reflecting mirror was damaged.

**Figure 5:**
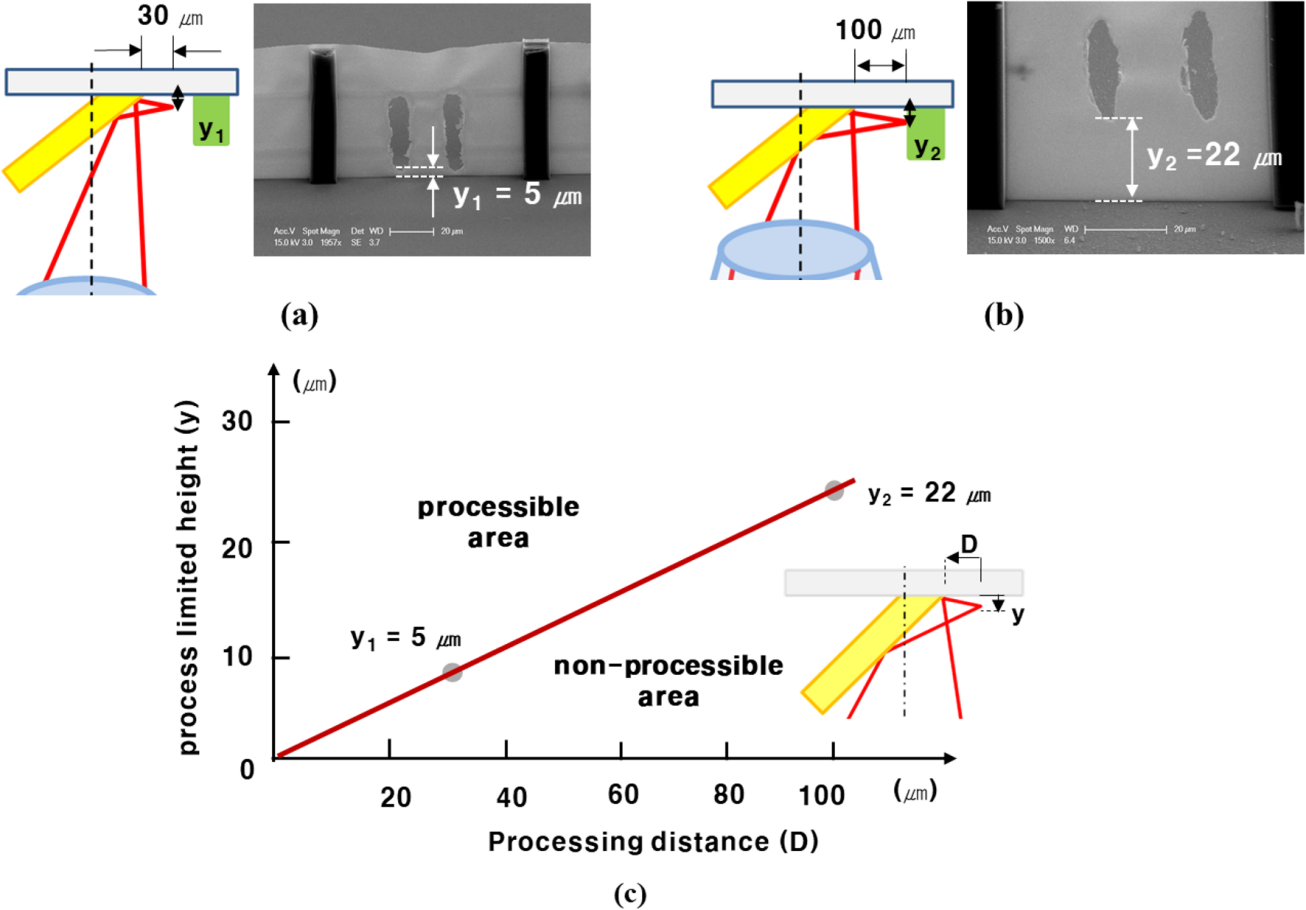
Schematic diagram and SEM image of multi-direction ablation process at process distance of (**a**) 30 μm and (**b**) 100 μm. The SEM images were measured with a 52° tilted SEM stage. The height was compensated according to 52° tilting. (**c**) The process limited the height according to the process distance based on the result from the SEM images.

As the reflecting mirror closely approached the microstructure, the ablation process-limited area was reduced. Figure 5a and b show that the ablation process limited the height of the microstructure when the laser was exposed to the lateral side of the microstructure and scanned using the galvano scanner. As shown in Fig. 5a, when the process distance is 30 μm, the height of the process is 5 μm. However, when the process distance was 100 μm from the target microstructure, the process-limited height increased to 22 μm. Because the process distance is far from the microstructure, the laser focus cannot reach the bottom of the microstructure, and the process-limited height is increased. When the processing distance is shortened, the process can be performed by minimizing the process-limited height. The process-limited height can be predicted using these experimental results (Fig. 5c).

### Experimental results with multi-directional ablation process

**Figure 6:**
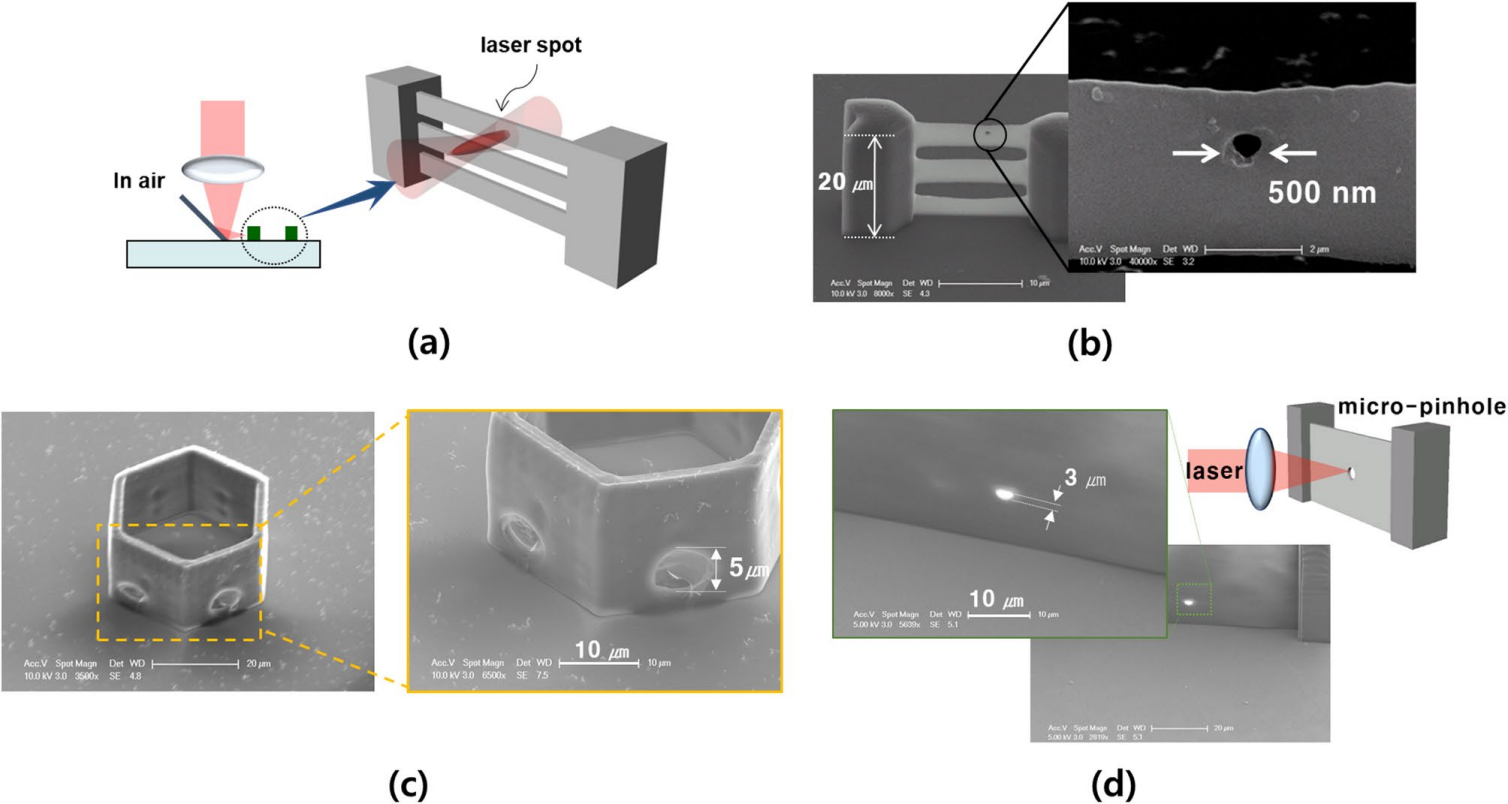
(**a**) Schematic illustration of the multi-direction ablation process. SEM images of the example fabrication by the process. (**b**) Single-point drilling on micro-beam. (**c**) Multi-point drilling on various processing planes. (**d**) Micro-pinhole structures for optical applications such as optical filters or diffraction patterns. The SEM images were measured with a 52° tilted SEM stage. The height was compensated according to 52° tilting.

For multi-directional ablation, the NA 0.3 objective lens was used. According to previous research, the optical shape of the focused laser is a prolate spheroidal shape^35^. A smaller NA resulted in an elongated focus shape. Moreover, the laser intensity along the travel direction is greater than that along the lateral direction^36^. The conventional laser ablation process is unsuitable for lateral surface patterning. For precise patterning on the lateral surface, the focal plane must be rotated using a multi-directional ablation system. As shown in Fig. 6a, when the multibeam structure needs to be drilled into a single-beam structure, the laser is reflected by the mirror, and the processing plane is rotated. This rotated focus leads to a precise hole pattern in a single-beam structure. A three-beam structure was fabricated using the TPS process. The height and width of each beam were 5 and 1 μm, respectively. To create a hole in the upper beam structure, the direction of the focal plane was precisely controlled using a reflecting mirror. To create a precise hole pattern, the laser power was 100 mW, which is the smallest laser power for the ablation process. As shown in Fig. 6b, a 500 nm single-hole pattern was formed in the upper beam structure. Figure 6c shows the multipoint drilling ability of a 3D microstructure by a multidirectional ablation process. The six-wall structure was fabricated by the TPS process, and the reflected and rotated laser focus was exposed to each wall using a z-axis piezo stage and a galvano scanner. Consequently, a 5 μm sized hole was selectively patterned on each six-wall structure. Therefore, the multidirectional ablation process allows the creation of a pattern on various surfaces of the microstructure for various applications.

The multidirectional ablation process is suitable for micro pinhole structures. The flat thin-wall was fabricated by TPS, and a micro-hole was drilled on the lateral side of the structure by a multi-directional ablation process (Fig. 6d). The fabrication time for the flat thin-wall was about 10 min, and the ablation time for drilling a micro-hole was within 1 ms. If the flat thin-wall with a micro-hole is fabricated by the TPS process, the fabrication time will increase for several minutes due to the additive fabrication. Therefore, the multi-directional ablation process is a very effective process from the point of view of fabrication time. These micro pin-hole structures can be used in various optical applications, such as optical filters and diffraction grids. Using this micro-pinhole structure, we demonstrated Twyman-Green interferometry on a microscale. Twyman-Green interferometry can be applied as a sensor to measure the travel distance of a sample. Micro lenses, micromirrors, and micro-prisms were fabricated using the TPS process. A micro-pinhole can be fabricated using a hybrid fabrication process with TPS and a multi-directional laser ablation process. Thus, hybrids of the TPS and multi-directional ablation process are expected to be applied in optical elements for various optical experiments on the microscale in the future.

## Conclusion

In this study, a multidirectional ablation process was proposed to create a pattern on the lateral side of a microstructure. It required a reflecting mirror system that could control the focal plane of laser ablation. The N.A. 0.3 objective lens was employed for this multi-directional ablation system considering the objective lens specifications such as the working distance, spot diameter, and pupil diameter. A reflecting mirror for the laser path guide was produced using MEMS. For high reflectivity, the reflecting mirror was coated with gold, and the angle of the tip of the reflecting mirror was 22 ° to reduce the interference between the reflecting mirror and substrate. Additionally, the range of the multi-directional ablation process was derived by considering the laser power and processing distance. Therefore, we derived a process range that does not damage the reflective mirror surface.

Various 3D microstructures were fabricated using the TPS process, and the lateral surface of the 3D microstructure was ablated using a multi-directional ablation process. This showed that the multidirectional laser ablation process could expand the processability of the microfabrication process for various microstructures in the future.

## Data Availability

All data generated or analyzed during this study are included in this published article. The datasets used and/or analyzed during the current study are available from the corresponding author on reasonable request.
